# An Effective Workflow for Differentiating the Same Genus Herbs of Chrysanthemum morifolium Flower and Chrysanthemum Indicum Flower

**DOI:** 10.3389/fphar.2021.575726

**Published:** 2021-04-22

**Authors:** Jiao He, Qian Zhang, Cuiying Ma, Gabriel I. Giancaspro, Kaishun Bi, Qing Li

**Affiliations:** ^1^ School of Pharmacy, Shenyang Pharmaceutical University, Shenyang, China; ^2^ Department of Dietary Supplements and Herbal Medicines, Science Division, United States Pharmacopeial Convention, Rockville, MD, United States

**Keywords:** *Chrysanthemum* morifolium flower, *Chrysanthemum* indicum flower, comparative evaluation, anti-inflammatory, phytochemical analysis

## Abstract

*C*. morifolium flower and *C. indicum* flower are two closely related herbal species with similar morphological and microscopic characteristics but are discriminated in edible and medicinal purpose. However, there is no effective approach to distinguish the two herbs. A novel workflow for quickly differentiating *C. morifolium* flower and *C. indicum* flower was developed. Firstly, the difference in anti-inflammatory effects for *C. morifolium* flower and *C. indicum* flower was characterized using lipopolysaccharide-treated rats. Then HPLC fingerprint analysis for 53 batches of *C. morifolium* flowers and 33 batches of *C. indicum* flower was carried out to deep profile the chemical components. The preliminary markers were screened out by OPLS-DA, identified by HPLC-ESI-QTOF-MS, and quantified by the improved SSDMC (single reference standard to determine multiple compounds) approach. Finally, multiple statistical data mining was performed to confirm the markers and a binary logistic regression equation was built to differentiate *C. morifolium* flower and *C. indicum* flower successfully. In general, the established workflow was rapid, effective and highly feasible, which would provide a powerful tool for herb identification.

## Introduction

*Chrysanthemum morifolium* flower [*Chrysanthemum x morifolium* (Ramat.) Hemsl.] and its wild relative, *Chrysanthemum indicum* flower (*Chrysanthemum indicum* L.), are commonly used as medicinal and edible cognate plants in Asia. *C. morifolium* flower and *C. indicum* flower have been widely used as food supplements, as well as herbal teas and health foods in China for 3,000 years (Lin and Harnly, 2010). Modern pharmacological studies indicate that both the two herbs possess various bioactivities, including anti-inflammation (Su et al., 2012; Han et al., 2015), antioxidation (Cui et al., 2012), cardiovascular protection (He et al., 2012), anticancer (Liu et al., 2018), etc.

In the Chinese Pharmacopoeia, *C. morifolium* flower is used for “scattering cold,” “cleaning heat and toxin,” and “brightening eyes,” and *C. indicum* flower is used to remove toxic heat (Chinese Pharmacopoeia Commission, 2020). Inflammation is the basic pathological changes of all these diseases. Inflammatory cytokines play an important role in the process, which is the interaction between pro-inflammatory cytokines like tumor necrosis factor-α (TNF-α), prostaglandin E2 (PGE2), interleukin (IL)-2, IL-6, IL-17, IL-23, and anti-inflammatory cytokines such as IL-4, IL-10, and IL-13 (Cheng et al., 2005). The dynamic change between pro-cytokines and anti-cytokines determines the ending and outcome of inflammation. Research on cytokines in inflammation especially in infective inflammation is meaningful as it may be the next breakthrough of a thorough cure in inflammatory disease (Gabay and Kushner, 1999; Kim et al., 2015). However, a comparative study of the anti-inflammatory activity of the two herbs remains to be investigated.

**FIGURE 1:**
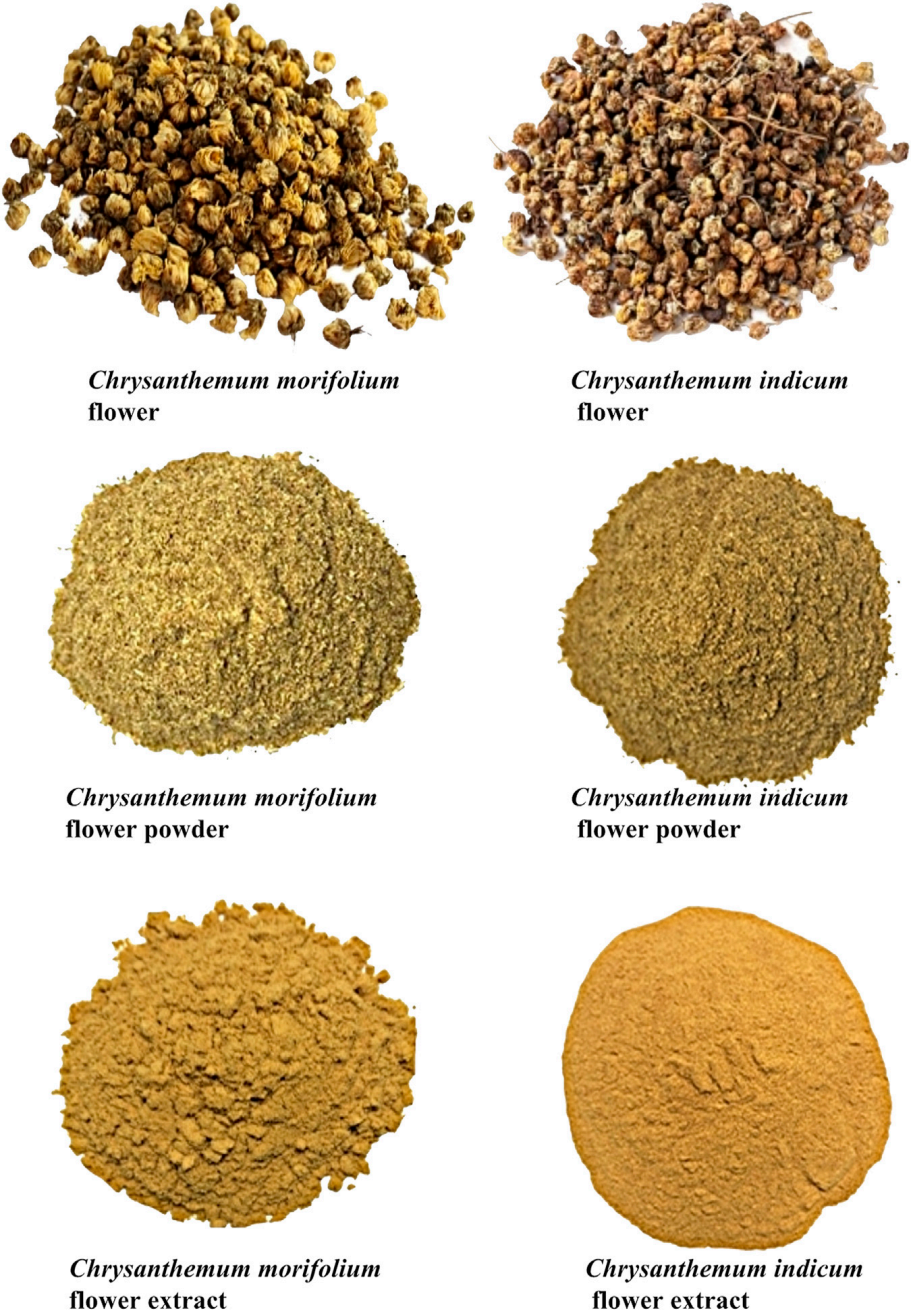
Representative photos of *C. morifolium* flower and *C. indicum* flower.

Though there are some differences between fresh *C. morifolium* flower and *C. indicum* flower, multiple batches of *C. morifolium* flower and *C. indicum* flower materials were easy to confuse due to different harvest periods, different origins, different processing or other reasons, especially for non-professionals (Fang et al., 2012; Japanese Pharmacopoeia Commission, 2016; Chinese Pharmacopoeia Commission, 2020). In addition, the commercialized products of *C. morifolium* flower and *C. indicum* flower are often sold in processed form as powder or extract (shown in Figure 1), which is more likely to lead to species misidentification and subsequent substitution. Furthermore*,* the two closely related herbal species have similar chemical compositions but are discriminated in medicinal and tea use due to differences in the contents of active compounds (Committee, C. P. 2015). However, the two herbs are usually assessed independently for quality using one or several marker compounds (Wu et al., 2010; Liu et al., 2013; Committee, C. P. 2015). The markers detected might be not sufficient to distinguish between herbal drugs that have similar appearances and/or chemical compositions (Osathanunkul et al., 2016). Therefore, the development of comparative quality evaluation and characterization methods for *C. morifolium* flower and *C. indicum* flower is essential.

**FIGURE 2:**
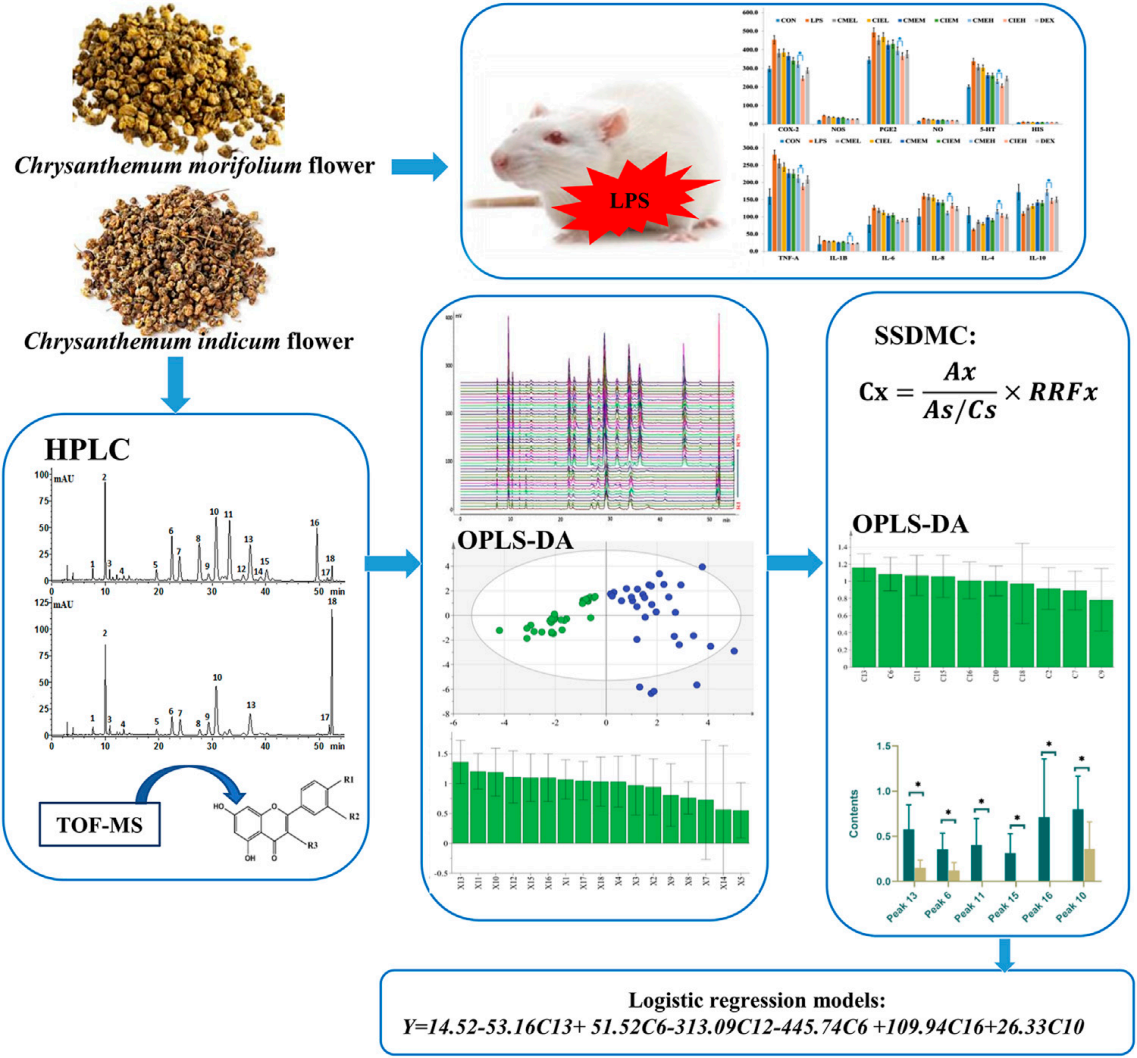
The graphic workflow of the paper.

Therefore, the present study aimed to define the chemical composition and pharmacological characteristics for comparative evaluation of *C. morifolium* flower and *C. indicum* flower (shown in Figure 2). First, the anti-inflammation activities of *C. morifolium* flower and *C. indicum* flower were systematically compared using lipopolysaccharide-treated rats. Then HPLC fingerprint analysis for 53 batches of *C. morifolium* flowers and 33 batches of *C. indicum* flower was carried out to deep profile the chemical components and differentiate the two herbs. The preliminary markers were screened out by OPLS-DA, identified by HPLC-ESI-QTOF-MS, and quantified by the improved SSDMC (single reference standard to determine multiple compounds) approach. Finally, multiple statistical data mining was performed to confirm the markers and a binary logistic regression equation was built to differentiate *C. morifolium* flower and *C. indicum* flower successfully.

## Materials and Methods

### Chemicals, Reagents and Materials

Isochlorogenic acid A, isochlorogenic acid B, isochlorogenic acid C, chlorogenic acid, luteolin-7-*O*-glucoside, apigenin-7-*O*-glucoside, linarin, luteolin-7-*O*-glucuronide, diosmetin-7-*O*-glucoside, caffeic acid, neochlorogenic acid and cryptochlorogenic acid (purity > 98%) were purchased from Chengdu Must Biological Technology Co. Ltd., (Chengdu, China). Water for HPLC was redistilled. Other chemical reagents were of HPLC grade. Dexamethasone sodium phosphate injection (DEX) (Specification: 1 ml: 2 mg) was purchased from Tianjin pharmaceutical Group Xinzheng Co. Ltd. Lipopolysaccharides (LPS) (*Escherichia coli* 055: B5) was purchased from Beijing Solaibao Biotechnology Co. Ltd., (Beijing, China). ELISA kits of rat 5-Hydroxytryptamine (5-HT), cyclooxygenase-2 (COX-2), Prostaglandin E2 (PGE2), nitric oxide (NO), nitric oxide synthase (NOS), tumor necrosis factor *α* (TNF-α), Histamine (HIS), interleukin 1β (IL-1β), IL-4, IL-6, IL-8, and IL-10 were purchased from Beijing Qisong Biotechnology Co. Ltd., (Beijing, China).

As listed in Table 1, 37 samples of *C. morifolium* flowers were purchased from herb markets, including the two cultivars of “*Gongju*” (from Huangshan City, Anhui Provence) and “*Hangbaiju*” (from Tongxiang County, Zhejiang Provence), which were considered as the genuine medicinal materials. From different farms owned by Zhejiang Conba Pharmaceutical Co. Ltd., 16 samples of *C. morifolium* flower were also collected. In addition, 33 batches of *C. indicum* flower samples were collected from different provinces in China as summarized in a previous study (He et al., 2016). All of these samples were authenticated based on analyses of microscopic and macroscopic characteristics by Professor Jia Ying and deposited at the Centre of Chinese Material Medica at Shenyang Pharmaceutical University.

**TABLE 1:** Sample information of *C. morifolium* flower and *C. indicum* flower samples.

Group	Sample no	Chinese name	Origins	Morphological characteristics	Similarity (Mean ± SD)
I	G1∼19	*Gongju*	Huangshan, Anhui	Flower	0.865 ± 0.031
	H1∼9	*Hangbaiju*	Tongxiang, Zhejiang	Flower	0.884 ± 0.035
	H10∼18	*Hangbaiju*	Tongxiang, Zhejiang	Flower buds	0.914 ± 0.032
II	K1∼16	*Hangbaiju*	Lanxi, Zhejiang, etc.	Flower	0.838 ± 0.142
III	Y1-33	*Yejuhua*	Anhuietc.	Flower or flower buds	0.654 ± 0.121^*^

I: *C. morifolium* flower samples which were Genuine medicinal materials and purchased online or from markets; II: *C. morifolium* flower samples collected from the five introduction districts and provided by Zhejiang Conba Pharmaceutical Co., Ltd; III: *C. indicum* flower samples collected from 13 provinces which were summarized in a previous study (*1*). The significant difference in similarities between the two herbs was statistically evaluated by the Student’s *t*-test (*p < 0.05).

### Apparatus and Chromatographic Conditions

HPLC analysis was performed using a Shimadzu 20A HPLC System (Shimadzu Corporation, Japan) composed of a binary solvent delivery system, an on-line degasser, an auto-sampler, a column temperature controller and a photodiode array detector coupled with Lab solution software. Additional different HPLC instruments were also used, including an Agilent 1260 HPLC system composed of a quaternary solvent delivery system, an on-line degasser, an auto-sampler, a column temperature controller and a photodiode array detector coupled with an analytical workstation. HPLC analysis was performed on a Luna C_18_ column (4.6 × 250 mm, 5 μm, Phenomenex Inc., CA, United States) with a sample injection volume of 10 μl. The detection wavelength was set at 327 nm, the flow rate was 1.0 ml min^−1^, and the column temperature was maintained at 25°C. The mobile phase consisted of a mixture of solution A (0.1% glacial acetic acid in water) and solution B (acetonitrile) along a linear gradient as follows: 0–10 min (10–18% B), 10–14 min (18–19% B), 14–20 min (19–19% B), 20–35 min (19–20% B), 35–40 min (20–22% B), 40–45 min (22–25% B), 45–55 min (25–35% B), 55–60 min (95–95% B), 60–65 min (10–10% B).

LC–MS^n^ analysis was performed on an Agilent 1260 HPLC system coupled with a Triple TOF™ 5600 (AB SCIEX, Foster City, CA) with an ESI interface. The mass range was set at *m/z* 50–1,500. The optimum parameters of the MS/MS detector were set as follows: the ion spray voltage was 5000 V for positive ion mode and −4500 V for negative ion mode, the ion source temperature was set at 500°C, ion source gas 1 was set at 50 psi, ion source gas 2 was set at 50 psi, the curtain gas was set at 30 psi, and the declustering potential was set as 90 V. Peak View® Software V. 2.2 was used for data collection and processing.

### Sample Preparation for Bioassay

Fifty grams of *C. indicum* flower/*C. morifolium* flower powder was refluxed with1250 ml of 75% EtOH for 2 h, and the extract solution was evaporated in vacuo to an adequate concentration. Then the extract powders were obtained with a freeze-drying method. The yields of *C. indicum* flower and *C. morifolium* flower extracts were 25 and 26%, respectively. For the vivo experiment on inflammatory response, both the *C. indicum* flower exact (CIE) and *C. morifolium* flower extract (CME) were suspended in 0.5% sodium carboxymethylcellulose at the concentration of 80, 40 and 20 mg/ml prior to use.

### Sample Preparation for Qualitative and Quantitative Analysis

The reference mixture solutions of chlorogenic acid (0.1782 mg/ml), luteolin-7-*O*-glucoside (0.1110 mg/ml), isochlorogenic acid B (0.1234 mg/ml), isochlorogenic acid A (0.1882 mg/ml), apigenin-7-*O*-glucoside (0.1338 mg/ml), isochlorogenic acid C (0.1122 mg/ml), and linarin (0.2458 mg/ml) were prepared in methanol. The mixed stock solutions were serially diluted to produce calibration standard solutions. All standard solutions were kept at 4°C.

Five hundred milligrams of *C. indicum* flower (No. Y29)/250 g *C morifolium* flower (No. H5) powder was accurately weighed and transferred into a flask, followed by the addition of 25 ml of 60% methanol and sonication for 30 min. The supernatant was collected and filtered through a 0.22 μm membrane for qualitative and quantitative analysis.

### Evaluation of the Anti-Inflammatory Activity *In Vivo*

Sprague Dawley (SD) rats (male, 16 weeks of age, 180–220 g) were provided by the Medical Experiment Animal Center of Shenyang Pharmaceutical University. They were housed under controlled temperature (23–25°C) and 12 h light/12 h dark cycle for a week before the experiment. Food and water were freely available. Experiments were conducted in accordance with the guidelines of the Guiding Principles for the Care and Use of Laboratory Animals approved by the Committee for Animal Experiments in Shenyang Pharmaceutical University.

For the experiment, rats were randomly assigned to nine groups (eight for each). CME (100, 200, and 400 mg/kg) and CIE (100, 200, and 400 mg/kg) were tube-fed for 7 days. The dose of linarin was selected based on previous report [20] and our preliminary study. The normal control, positive control group (DEX) and control group (LPS) were given equal amount of vehicle during this period. 0.5 h after final administration, all of the animals except for rats in normal control groups were injected intraperitoneally with 100 μg/kg LPS to induce acute inflammation. Rats in positive control group were injected with DEX (50 μg/kg) 0.5 h before LPS challenge. All of the animals were anesthetized with pentobarbital sodium and then the blood samples were collected from the abdominal aorta at 6 h after LPS injection. Blood samples were immediately centrifuged at 3,000 rpm for 10 min. Sera were collected, frozen, and kept at −80°C until analysis.

### Calculation of Relative Conversion Factors in SSDMC Approach

According to the HPLC test results for the reference compound mixture solutions obtained using the method described in *Apparatus and Chromatographic Conditions*, the relative response factor (RRF_X_) could be calculated using the ratio of the peak areas and the ratio of the concentration of the analyte and internal reference substance (Eq. 1) (Hou et al., 2014):

$$\text{RRR}x=\frac{\sum^{\text{​}}\left(\frac{Asi}{Csi}\right)/\left(\frac{Axi}{Cxi}\right)}{N}\;\;i=1∼n,$$ (1)

where *Asi* and *Axi* are the peak areas of the internal reference substance and analyte, respectively, at the concentration level *i*. The variables *Csi* and *Cxi* are the concentrations (at level *i*) of the internal reference substance and analyte, respectively. N represents the number of concentration levels, which was seven in this work.

With the results of RRFx, the concentration of analyte (C_x_) in the samples was calculated based on the following equation:

$$Cx=\frac{Ax}{As/Cs}\times RRFx,$$ (2)

where *Ax* and *As* are the peak areas of the analyte and reference component, respectively. *Cx* and *Cs* are the concentrations of the analyte and reference compound in the *sample solution* and *standard solution*, respectively. RRFx is the relative response factor of the analyte vs. the reference compound.

### Validation of the Quantitative Analysis Method

Analytical method validation ensures the suitability and ruggedness of the method as a quality measure for use across multiple laboratories. The method developed for quantitative analysis of the major caffeoylquinic acids and flavone glycosides in *C. morifolium* flower and *C. morifolium* flower was validated by tests of linearity, limits of detection (LODs), limits of quantification (LOQs), accuracy, precision (intra- and inter-day variability), robustness and stability.

## Results and Discussion

### Anti-inflammatory Activity of C. morifolium Flower and C. indicum Flower Extract in Lipopolysaccharide-treated Rats

Gram-negative bacterial endotoxins or LPS, are associated with tissue injury and fatal outcome in septic shock (Girish, 2013). It has been demonstrated both experimentally and clinically that sepsis causes the production of a series of proinflammatory cytokines (IL-1β, TNF-α, IL-8, and IL-6), anti-inflammatory cytokines (IL-4 and IL-10), inflammatory mediums (NO, HIS, 5-HT, PGE_2_), and related enzymes (iNOS, COX-2), which determine the ending and outcome of inflammation (Gouwy et al., 2005; Chen et al., 2018; Dickson and Lehmann, 2019).

**FIGURE 3:**
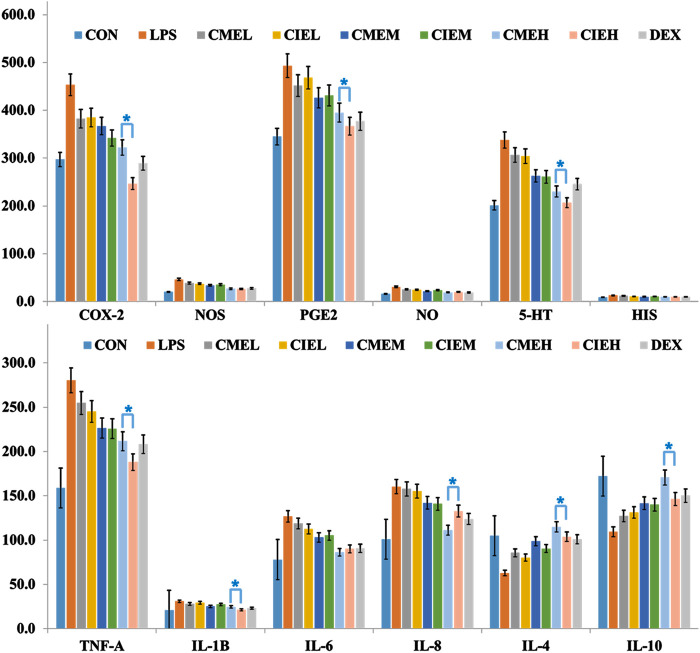
Anti-inflammation effect of *C. morifolium* flower extract (CME) and *C. indicum* flower extract (CIE) in lipopolysaccharide-induced rats. The data are expressed as the mean ± S.D. The significant difference between groups of CME and CIE with different dose was statistically evaluated, respectively, by the Student’s *t*-test (*p < 0.05).

In the present study, levels of serum inflammatory cytokines, inflammatory mediums and related enzymes were measured by ELISA at 6 h after LPS injection based on pilot experiments results and the previous reports who demonstrated peaks in serum cytokines concentrations at 4–6 h after LPS injection (Kim et al., 2014). As shown in Figure 3, ELISA results displayed that all extracts of *C. morifolium* flower and *C. indicum* flower can cause a dose-dependent decrease in pro-inflammation cytokines, inflammatory mediators and related enzymes, and a dose-dependent increase in anti-inflammatory cytokines for anti-inflammatory effects. Moreover, high dose groups (400 mg/kg) of *C. morifolium* flower and *C. indicum* flower exhibited significant difference in inhibition or promotion of inflammatory mediums (NO, HIS, 5-HT, PGE_2_), and related enzymes (iNOS, COX-2) serum cytokines (*p* < 0.05), while low (100 mg/kg) and moderate (200 mg/kg) dose groups of *C. morifolium* flower and *C. indicum* flower showed no significant variation was observed between each other. Comparatively, high dose group of *C. indicum* flower reflected stronger inhibitory effects for COX-2, PEG2, 5-HT, TNF-α, IL-β in serum. While, high dose group of *C. morifolium* flower appeared to show stronger promoting effects for IL-4 and IL-10. In addition, all the groups with the same dose of *C. morifolium* flower and *C. indicum* flower showed no significant difference in expression of NOS, NO, HIS, and IL-6.

### HPLC Fingerprint Analysis

**FIGURE 4:**
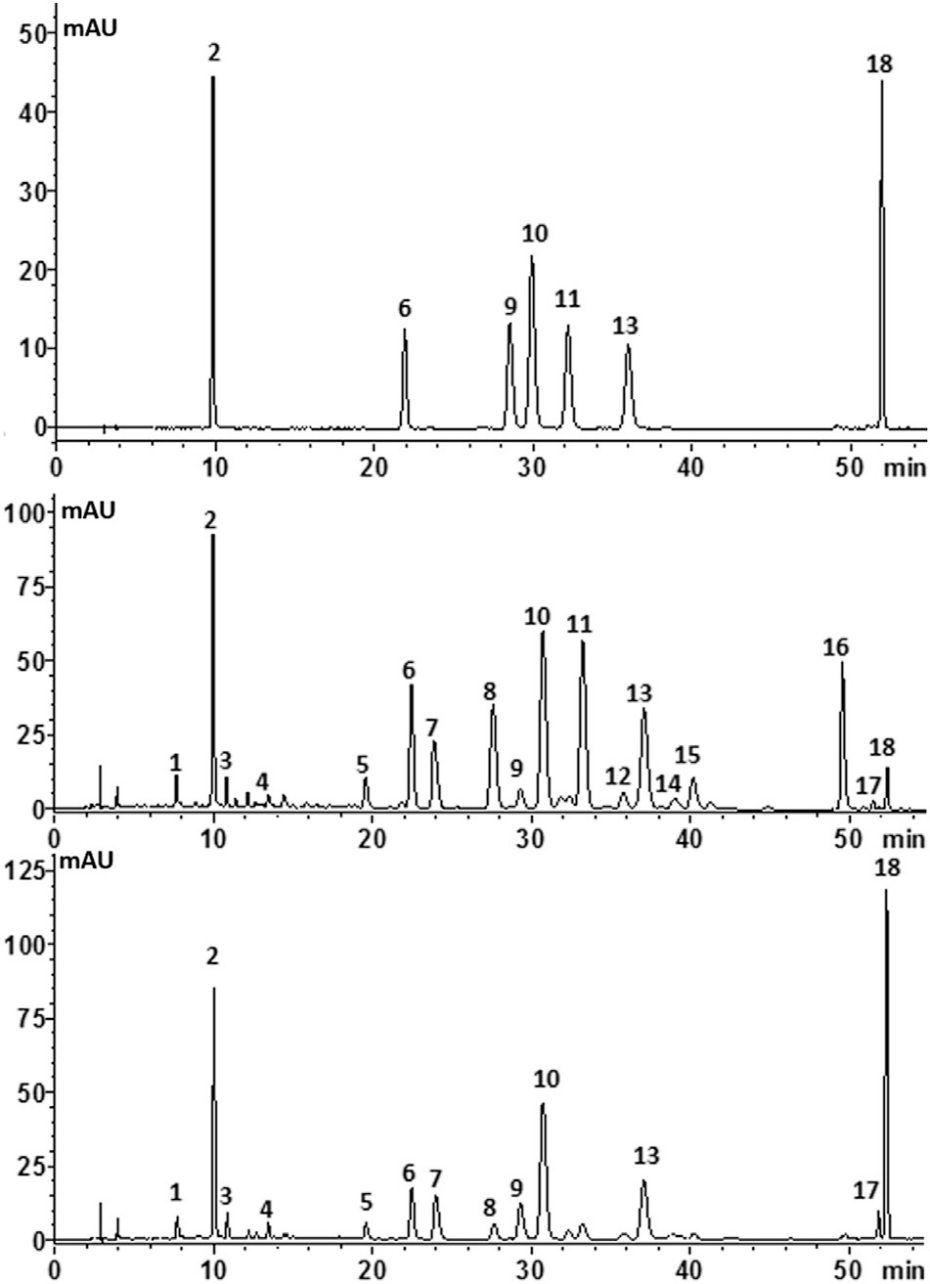
Typical chromatograms of **(A)** mixed standard solution, **(B)** *C. morifolium* flower and **(C)** *C. indicum* flower with the compounds numbered as follows: (1) neochlorogenic acid; (2) chlorogenic acid; (3) cryptochlorogenic acid; (4) caffeic acid; (5) luteolin-7-O-rutinoside; (6) luteolin-7-*O*-glucoside; (7) luteolin-7*-O-* glucuronide; (9) isochlorogenic acid B (10) isochlorogenic acid A (11) apigenin-7*-O-*glucoside; (13) isochlorogenic acid C; (15) diosmetin-7-*O*-glucoside; (16) apigenin-7-*O*-6″-malonylglucoside (18) linarin.

In the present work, the HPLC fingerprint analysis was validated and considered to be effective and reliable. Then, 53 batches of *C. morifolium* flower (“*Hangbaiju*” and “*Gongju*”) and 33 batches of *C. indicum* flower samples from different regions (Table 1) were analyzed under the optimized HPLC conditions. Figure 4 shows typical fingerprinting chromatograms of *C. morifolium* and *C. indicum* flowers. Eighteen common peaks, which moderately existed in all chromatograms from the samples and indicated similarity among the various samples, were collected from *C. morifolium* flower samples by comparison of the UV spectra and retention times. Chromatograms of *C. indicum* flower confirmed the abundance of peak 18 but showed a deficiency of peaks 11, 12, 14, 15, and 16 compared with the spectra of *C. morifolium* flower samples.

CFDA suggests that all herbal chromatograms should be evaluated in terms of similarity based on a calculation of the correlation coefficient and/or angle cosine value of the original data (Zhao et al., 2011). The similarities (Table 1) between the mean chromatogram (both *C. morifolium* flower and *C. indicum* flower) and each herb sample were within a range of 0.341–0.805 (*C. indicum* flower) and 0.816–0.947 (*C. morifolium* flower). These preliminary examinations showed that it was possible to discriminate *C. morifolium* and *C. indicum* flower samples by calculating the correlation coefficients of the main secondary metabolite profiles. It also demonstrated that *C. indicum* flower samples from numerous wild regions had a large fluctuation in quality, while the chromatograms of *C. morifolium* flower samples considered to be genuine were comparatively stable and consistent. In addition, *C. morifolium* flower (“*Hangbaiju*”) samples from Zhejiang Province (samples considered to be genuine) had higher correlation coefficients of similarity (0.833–0.947), while samples (K10-15) from Jiangsu and Hubei Provinces (introduction areas) had lower correlation coefficients of similarity (0.624–0.886). The results indicated that there were differences in the internal quality of “*Hangbaiju*” between samples grown in Zhejiang and those grown in the other two provinces of Jiangsu and Hubei.

**FIGURE 5:**
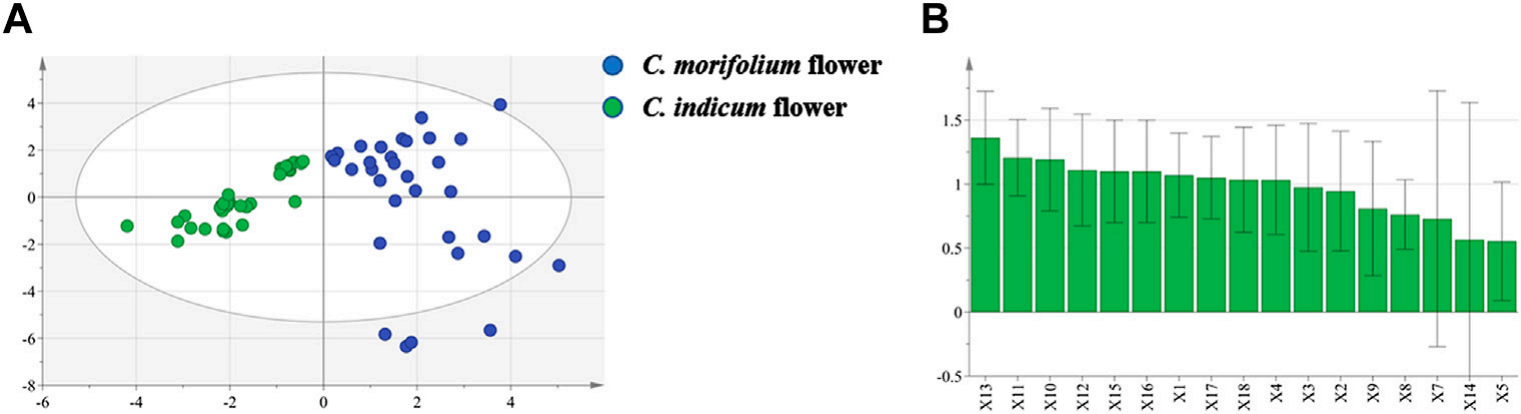
OPLS-DA projection plots in HPLC fingerprint analysis: **(A)** Score plot of 33 samples of *C. indicum* flowers (CI) and 53 samples of *C. morifolium* flowers (CM) from different origins; **(B)** *VIP* values of the components in differentiating CM and CI.

OPLS-DA was used to preliminarily screen out the markers for differentiating *C. morifolium* and *C. indicum* flower samples based on the relative peak areas (RPAs) of common peaks. The score plot (Figure 5A) showed that *C. morifolium* flower and *C. indicum* flower were separated clearly (*R* ^*2*^ *X* 0.736, *R* ^*2*^ *Y* 0.756, *Q* ^*2*^ 0.725). Combined *VIP* value (Figure 5B) of OPLS-DA (*VIP* > 1.0) and *p* value of Student’s *t*-test (*p* < 0.05), potential marker pool was generated, which contained peaks 13, 11, 10, 12, 15, 16, 1, 17, 18, 4.

### Identification of Potential Markers Between *C. morifolium* and *C. indicum* Flowers

Based on HPLC-DAD and HPLC-ESI-QTOF-MS analysis, 14 detected peaks in *C. morifolium* and *C. indicum* flowers were identified or tentatively characterized using the chemical reference standards by comparing UV spectra, retention times, precursors and fragment ion (*m/z*) values. The UV spectrums and extracted ion chromatograms of CM and CI were shown in Supplementary Figures S1, S2 in Supporting Information. The structures and detailed MS data of the identified components were summarized in Table 2. Compounds 1, 2 and 3 yielded [M-H]^−^ peaks at *m/z* 353.0876 (calculated for C_16_H_18_O_9_, 353.0873) and fragment ions at *m/z* 191.0559 due to the cleavage of the caffeoyl band, suggesting that monocaffeoylquinic acids were present. Compounds 9, 10, and 13 showed [M-H]^−^ peaks at *m/z* 515.1645 (calculated for C_25_H_24_O_12_, 353.0873), which produced two successive neutral losses from caffeic acid that yielded two stable fragment ions at 353.0845 [M-H-C_9_H_6_O_3_]^−^ and 191.0536 [M-H-2C_9_H_6_O_3_]^−^, suggesting that dicaffeoylquinic acids were also present. Compound 4 produced [M-H]^−^ peaks at *m/z* 179.0351 (calculated for C_9_H_8_O_4_), which produced a stable ion fragment at 135.0451 [M-H-CO_2_]^−^, suggesting that this compound was caffeic acid. Then, the seven compounds numbered 1, 2, 3, 4, 9, 10, and 13 were unambiguously identified as neochlorogenic acid, chlorogenic acid, cryptochlorogenic acid, caffeic acid, isochlorogenic acid B, isochlorogenic acid A, and isochlorogenic acid C, respectively, based on comparison of the experimental retention times, UV spectra and MS^n^ data with those of the standards.

**TABLE 2:** Characterization of constituents by LC-MSn analysis in negative-ion modes.

No	t_R_ (min)	Formula	Mass error (ppm)	Fragment ion MS (*m/z*) in negative ion mode	Identification	Structural formula		
**Caffeoylquinic acid and caffeic acid**						**R_3_**	**R_4_**	**R_5_**
1	7.03	C_16_H_18_O_9_	−1.8	353.0871[M-H]^−^, 91.0559, 135.0452	Neochlorogenic acid	H	H	*C*
2	9.89	C_16_H_18_O_9_	−3.3	353.0866[M-H]^−^, 191.0562	Chlorogenic acid	*C*	H	H
3	10.24	C_16_H_18_O_9_	−1.7	353.0875[M-H]^−^, 191.0562	Cryptochlorogenic acid	H	*C*	H
4	12.14	C_9_H_8_O_4_	0.8	179.0351[M-H]^−^, 135.0451	Caffeic acid			
9	23.96	C_25_H_24_O_12_	−3.6	515.1175[M-H]^−^, 353.0871, 191.0558, 135.0455	3,4-Dicaffeoylquinic acid	*C*	*C*	H
10	26.28	C_25_H_24_O_12_	−3.3	515.1177[M-H]−, 353.0853, 191.0544, 135.0446	3,5-Dicaffeoylquinic acid	*C*	H	*C*
13	32.22	C_25_H_24_O_12_	−3.0	515.1179[M-H]^−^, 353.0845, 191.0536	4,5-Dicaffeoylquinic acid	H	*C*	*C*
**Flavonoid glycoside**						**R_7_**	**R_3’_**	**R_4’_**
5	18.86	C_27_H_30_O_15_	−1.0	593.1496[M-H]^−^ 447.0950[M-H-rhamnose]^−^, 285.0393 [M-H-rutinoside]^−^	Luteolin-7*-O-*rutinoside	−Rutinose	OH	OH
6	20.45	C_21_H_20_O_11_	1.0	447.0911[M-H]^−^ 285.0393[M-H-glucose]^−^	Luteolin-7*-O-*glucoside	−Glucoside	OH	OH
7	21.13	C_21_H_18_O_12_	−3.4	461.0712[M-H]^−^ 357.0614[M-H-rutinoside]^−^ 285.0380[M-H-glucuronide]^−^	Luteolin-7*-O-*glucuronide	−Glucuronide	OH	OH
11	29.01	C_21_H_20_O_10_	−2.2	431.0954[M-H]^−^ 269.0437[M-H-glucose]^−^	Apigenin-7*-O-*glucoside	−Glucose	H	OH
15	38.12	C_22_H_22_O_11_	−3.8	461.1067[M-H]^−^ 299.0540[M-H-glucose]^−^ 284.0300[M-H-glucose-CH3]−	Diosmetin-7*-O-*glucoside	−Glucose	OH	OCH_3_
16	45.96	C_24_H_22_O_13_	0.2	517.0945[M-H]^−^ 473.1068[M-H-CO2l]− 431.0970[M-H-malonyl]^−^ 269.0437[M-H-malonyl-glucose]^−^	Apigenin-7*-O-*(6″-malonyl)glucoside	−(6″-malonyl) glucose	H	OH
18	52.45	C_27_H_30_O_15_	0.1	591.1681[M-H]^−^ 283.0586[M-H-rutinose]^−^ 268.0350[M-H-rutinose-CH3]	Linarin (acacetin-7*O-*rutinoside)	−Rutinose	H	OCH_3_

Seven major flavonoids were identified in the two herbal medicines, five of which (peaks 6, 7, 13, 15, and 18) were unambiguously identified by comparing the obtained data with those from the reference standards. The produced ions [M-H-308]^−^ at *m/z* 287.0393 ([M-H-glucose]^−^) from compound 5 [M-H-162]^−^ at *m/z* 287.0393 ([M-H-glucoside]^−^) from compound 6, and [M-H-176]^−^ at *m/z* 285.0380 ([M-H-glucuronide]^−^) from compound 7 were indicative of the glucuronide, glucoside and glucuronide of luteolin, respectively. Compound 11 produced an [M-H]^−^ ion at *m/z* 431.0954 [M-H]^−^ (calculated for C_21_H_20_O_10_, 465.1033), and moderately abundant [M-H-162]^−^ product ions at *m/z* 269.0437 were formed through the neutral losses of glucose, indicating that this compound was apigenin-7*-O-*glucoside. Compound 16 produced [M-H-248]^−^ and [M-H-86]^−^ fragments due to the loss of malonyl-glucose and malonyl (-COCH_2_COO-) from their molecular ions, suggesting that this compound was the malonyl glucoside of apigenin. The fragment ions [M-H-162]^−^ at *m/z* 299.0540 produced from compound 15 were indicative of the glucosides of diosmetin, and the fragment ions at *m/z* 284.0300 were formed by the neutral losses of CH_3_. The fragment ions ([M-H-rutinoside]^−^) at *m/z* 283.0586 from compound 18 were indicative of the rutinoside of acacetin. The fragment ion at *m/z* 151.0021 yielded through the loss of CH_3_ showed the existence of a 3′-OCH_3_ group. In general, these compounds were identified as glycosylated derivatives of apigenin, luteolin, diosmetin and acacetin.

### Quantitative Analysis of Potential Markers Between *C. morifolium* and *C. indicum* Flowers

Considering that all potential markers between *C. morifolium* and *C. indicum* flowers belong to caffeoylquinic acids (Peak 13, 10, 1, and4) or flavone glycosides (Peak 11, 15, 16, and 18), quantitative analysis was conducted for the two types of compounds in the tested samples by improved SSDMC and standard calibration methods.

#### Calculation of Relative Response Factors and Relative Retention Times

The SDDMC method was mainly developed to simultaneously identify a group of compounds with similar polarity characteristics, UV spectra and chromatographic behaviors (Wang et al., 2015). In this work, chlorogenic acid and luteolin-7-*O*-glucoside, which were stable, accessible, and abundant in the samples, were selected as internal reference substances to determine the other three caffeoylquinic acids (isochlorogenic acid A, isochlorogenic acid B, and isochlorogenic acid C) and two flavone glycosides (apigenin-7-*O*-glucoside and linarin). Based on the series of standard solutions, the final RRF of each analyte was calculated using the average of several RRFs detected from the series of concentrations and calculated according to Eq. 1. The calculation of RRT was necessary to identify the peaks using only the internal standard for the SSDMC method. To develop an SSDMC method with general applicability, it was essential to evaluate the ruggedness of the RRT and RRF on different columns (different batches of Luna C_18_) and equipment. As summarized in Table 3, the RRFs for each analyte were quite similar at the detection wavelength of 327 nm across different HPLC instruments, indicating a good consistency of the RRFs. The RRT for each analyte was stable with the RSDs, with deviations of less than 2.0%, and was found to be suitable for use in the identification.

**TABLE 3:** Ruggedness of the RRT and RRF of marker components in C. morifolium/C. indicum flower, *n* = 7.

Analyte	Shimadzu20 A		Agilent1260		RSD (%)	
	RRT	RRF	RRT	RRF	RRT	RRF
Chlorogenic acid^a^	1.00	-	1.00	-	-	-
Isochlorogenic acid B^a^	2.89	0.93	2.90	0.93	0.2	1.4
Isochlorogenic acid A^a^	3.26	0.83	3.27	0.82	0.2	1.6
Isochlorogenic acid C^a^	3.84	0.89	3.85	0.88	0.2	0.8
Luteolin-7*-O-*glucoside^b^	1.00	-	1.00	-	-	-
Apigenin-7*-O-*glucoside^b^	2.89	0.76	2.90	0.77	0.2	1.0
Linarin^b^	3.26	1.04	3.25	1.04	0.2	3.1
Luteolin-7*-O-*glucuronide^b^	1.04	1.03	1.05	1.03	0.7	-
Diosmetin-7*-O-*glucoside^b^	1.65	1.03	1.64	1.03	0.4	-
Apigenin-7*-O-*6″-malonylglucoside^b^	2.65	0.91	2.64	0.91	0.3	-

Components were identified and quantified using chlorogenic acid.

^a^ or luteolin-7*-O-*glucoside.

^b^ as internal reference substances.

In addition, previous studies showed that the RRFs of flavones presenting the same or similar skeletons and different substituent groups were correlated with molecular weights (Cui et al., 2016). Due to a lack of reference standards, the RRFs of the three flavone glycosides luteolin-7*-O-*glucuronide, diosmetin-7-*O*-glucoside and apigenin-7-*O*-6″- malonylglucoside were calculated based on analytes with highly similar molar absorptivities and molecular weights. For example, the structures of luteolin-7*-O-* glucuronide (Mr = 462.3) and diosmetin-7-*O*-glucoside (Mr = 462.3) were similar to that of luteolin-7-*O*-glucoside (Mr = 448.3). Thus, the RRFs of the two analytes were both obtained as 1.03 (RRF = 462.3/448.3 × 1.00). The RRF of apigenin-7-*O*-6″- malonylglucoside (Mr = 518.1), which possessed a structure similar to that of apigenin-7-*O*-glucoside (Mr = 432.8), was determined to be 0.91 (RRF = 518.1/432.8 × 0.76). Though the results of the three RRFs may deviate from the true value and are only an approximation, the method was validated as an effective alternative for the quality control of *C. morifolium* flower and *C. indicum* flower when lacking of the sufficient chemical standard substances.

#### Validation of the Quantitative Analysis Method

For the ESM method, all of the reference standards solutions related to the analytes to be examined should be prepared first. In contrast, only the internal reference solutions were needed for the SSDMC method. The results calculated via the SSDMC method in this experiment were compared to the results obtained with ESM (shown in Supplementary Tables S1–S8).

The linearity equation was constructed using a series of standard solutions. The calibration curves calculated by plotting the peak area *Y* against the concentration *x* (μg/ml) of each compound were *Y*_*1*_ = 2.564×10^4^ *x* + 5.208 × 10^3^, *Y*_*2*_ = 2.006 × 10^4^ *x* + 0.745 × 10^3^, *Y*_*3*_ = 2.756 × 10^4^ *x* + 1.954 × 10^3^, *Y*_*4*_ = 3.129 × 10^4^ *x* + 0.973 × 10^3^, *Y*_*5*_ = 2.628 × 10^4^ *x* + 7.102 × 10^3^, *Y*_*6*_ = 2.918 × 10^4^ *x* + 3.478 × 10^3^, and *Y*_*7*_ = 1.918 × 10^4^ *x* + 7.238 × 10^3^ for Peak 2, 9,10, 13, 6, 11, and 18, respectively. The calibration curves exhibited good linearity (*r* ^2^ > 0.9993) within the test range. The LOD (S/N = 3) was 0.49–0.79 ng/ml and the LOQ (S/N = 10) was 1.64–2.65 ng/ml for the seven compounds.

Repeatability was assessed by examining three replicate solutions prepared at three different concentrations (high, medium and low). The *RSD* values for the seven components in samples (H5, Y29) were less than 5.4%. The precision of the method was analyzed by using different operators and performing the analysis on different days, equipment and columns. The *RSD* values were found to be in the range of 1.2–4.2% (in S3–S7 in Supporting Information). The results showed no remarkable differences between the precision of the two methods, SSDMC and ESM, according to the F-test (*p* = 0.113 > 0.05).

The method accuracy was determined using a recovery test by assaying the known added amount of analyte in the sample at three concentration levels (75, 100, and 125%). Three replicates of each concentration in samples (H5, Y29) were examined. Recoveries were in the range 95.0–102.0% with *RSDs* less than 3.2% for the seven analytes in the samples (H5, Y29) (shown in S8-S9 in Supporting Information). The recoveries between the ESM and SSDMC methods showed no remarkable differences using the paired *t*-test (*p* = 0.174 > 0.05).

The stability of the sample solutions (H5, Y29) was examined by comparing the peak areas of the same sample solutions after storage for different times. The results demonstrated that the sample solutions were stable for almost 24 h with the *RSD*s of peak areas less than 2.6%.

### Workflow for Differentiating *C. morifolium* Flower and *C. indicum* Flower

Four caffeoylquinic acids (Peak 2, 9, 10, and 13) and three flavone glycosides (Peak 6, 11, and 18), in the tested samples were simultaneously determined by SSDMC and standard calibration methods. The quantitative results from the two methods were accordant using the *t*-test (*p* = 0.376, *p* > 0.05). Meanwhile, the other three flavone glycosides (Peak 7, 15, and 16) were determined by the RRFs calculated as described in *Calculation of Relative Response Factors and Relative Retention Times*. All of the results are summarized in Supplementary Tables S9–S11 in the Supporting Information. Contents of the ten components in the different samples varied greatly, with the total contents of four caffeoylquinic acids ranging from 0.684 to 3.445% in *C. morifolium* flower and 0.166–2.112% in *C. indicum* flower, while the total contents of six flavone glycosides ranged from 0.315 to 4.161% in *C. morifolium* flower and 0.144–2.078% in *C. indicum* flower. The average amount of Peak18 (linarin, 0.636%) in *C. indicum* flower was much higher (approximately six-fold) than that in *C. morifolium* flower but varied significantly among the different samples due to the numerous regions of origin and the variation of wild resources. In contrast, the other five flavone glycosides were detected at significantly lower levels in *C. indicum* flower. Furthermore, the absolute contents of the four caffeoylquinic acids in *C. morifolium* flower samples were significantly higher than those in *C. indicum* flower samples, but the content ratios of the four caffeoylquinic acids were similar in both *C. morifolium* flower and *C. indicum* flower.

**FIGURE 6:**
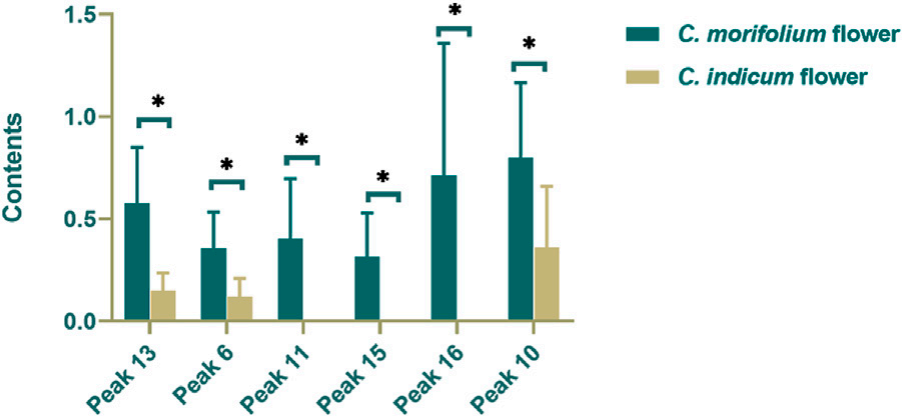
Contents of markers in *C. indicum* flower (CI) and *C. morifolium* flower. (*p < 0.05).

Based on the quantitative results, Student’s *t*-test and OPLS-DA (*R* ^*2*^ *X* 0.822, *R* ^*2*^ *Y* 0.764, *Q* ^*2*^ 0.715) were used to further screen out markers for differentiating *C. morifolium* flower and *C. indicum* flower. Combined *VIP* value and *p* value, peaks 13, 16, 11, 15, 16 and 10 were identified as the markers, whose contents were shown as Figure 6. Then to effectively distinguish the two hers from the same genus, a binary logistic regression equation was established as *Y* = 14.52 − 53.16*C13* + 51.52 *C* ^*6*^ − 313.09*C11* − 445.74 *C6* + 109.94*C16 + 26.33C10* (*C*x represents the content of peak x)*.* When the contents of the six markers were substituted into the equation, the sample was determined as *C. morifolium* flower if the result was negative and on the contrary for *C. indicum* flower. The established method was tested with multiple batches of samples collected from different regions, and the accuracy ratewas 100%, which proved the robust of the model.

## Conclusion

In summary, we demonstrated a systematic study for comparing *C. morifolium* flower and *C. indicum* flower from chemical composition to pharmacological characteristics. The difference in anti-inflammatory activity between the two herbs was firstly characterized. Potential markers for distinguishing *C. morifolium* flower from *C. indicum* flower were preliminarily screened out by HPLC fingerprint analysis combined with statistical methods and identified by HPLC-ESI-QTOF-MS. And improved SSDMC approach was used for quantifying the potential markers of four caffeoylquinic acids and six flavone glycosides. Finally, a binary logistic regression equation based on the contents of markers was built to differentiate *C. morifolium* flower and *C. indicum* flower successfully. The workflow for differentiating *C. morifolium* flower and *C. indicum* flower was effective and would provide a powerful tool for herb identification.

## Data Availability

The raw data supporting the conclusion of this article will be made available by the authors, without undue reservation, to any qualified researcher.

## Abbreviations

5-HT, 5-hydroxytryptamine; CFDA, china food and drug administration; CIE, *Chrysanthemum indicum* flower exact; CME, *Chrysanthemum. morifolium* flower extract; COX-2, cyclooxygenase-2; DEX, dexamethasone sodium phosphate injection; ESM, external standard method; HIS, histamine; HPLC-DAD, high performance liquid chromatography diode-array detector; HPLC-ESI-QTOF-MS, high performance liquid chromatography-electrospray ionization-quadrupole tandem time-of-flight mass spectrometry; IL-1β (IL-4, IL-6, IL-8, IL-10), interleukin 1β (interleukin 4, 6, 8, 10); LOD, limits of detection; LOQ, limits of quantification; LPS, lipopolysaccharides; NO, nitric oxide; NOS, nitric oxide synthase; OPLS-DA, orthogonal partial least squares discrimination analysis; PGE2, prostaglandin E2; RRF, relative response factor; RRT, relative response time; SSDMC, single reference standard to determine multiple compounds; RSD, relative standard deviation; TNF-α, tumor necrosis factor-α; UV, ultraviolet.
