# Supplementary material for: An Effective Workflow for Differentiating the Same Genus Herbs of Chrysanthemum morifolium Flower and Chrysanthemum Indicum Flower
Source: Front Pharmacol. 2021 Apr 22;12:575726. doi: 10.3389/fphar.2021.575726 (PMC8102030; doi:10.3389/fphar.2021.575726)
Supplement: Supplementary file 1 [file datasheet1.docx]

Supplementary Material

**An effective workflow for differentiating the same genus herbs of** ***Chrysanthemum morifolium* flower and *Chrysanthemum indicum* flower**

**Jiao He****^1^, Yiyang Du^1^, Cuiying Ma^2^,** **Gabriel I. Giancaspro^2^, Kaishun Bi^1^, Qing Li^1*^**

**^1^**School of Pharmacy, Shenyang Pharmaceutical University, Shenyang 110016, China

^2^Department of Dietary Supplements and Herbal Medicines, Science Division, U.S. Pharmacopeial Convention, 12601 Twinbrook Parkway, Rockville, MD 20852, USA

*** Correspondence:**
Corresponding Author
[lqyxm@hotmail.com](mailto:lqyxm@hotmail.com)

**1. Supplementary Figures**


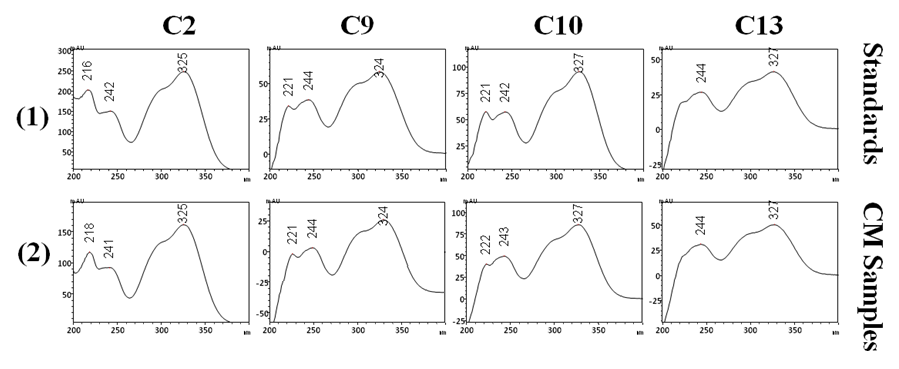


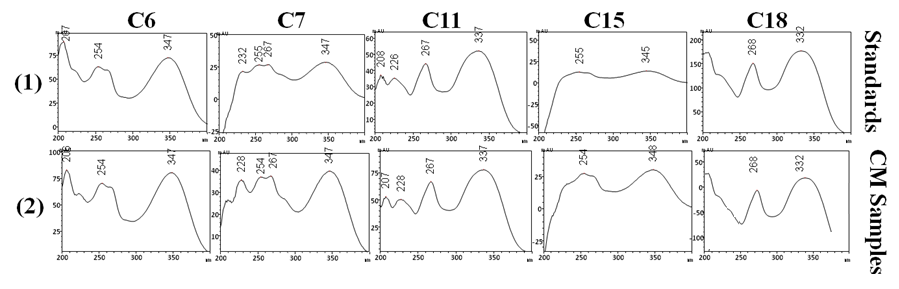


**(4)**

**(3)**

**Supplementary Figure 1.** UV spectrum of standards (1, 3) and analytes (2, 4) in sample solution of *C. morifolium* flower.


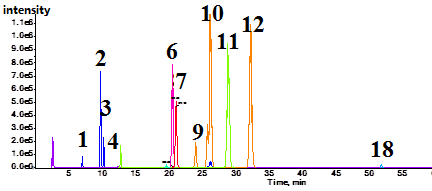


**(A)**


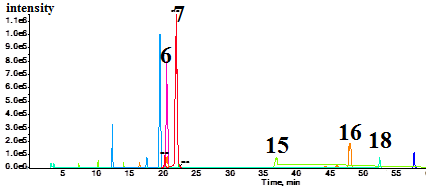


**(B)**


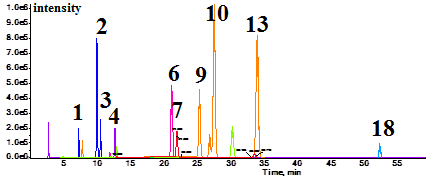


**(C)**


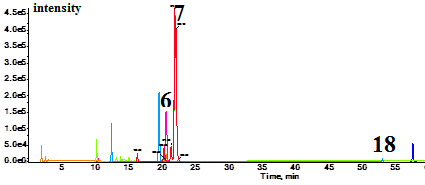


**(D)**

**Supplementary Figure 2** Extracted ion chromatograms of *Chrysanthemum morifolium* flower in negative mode (A) and positive ion mode (B); *Chrysanthemum indicum* flower in negative ion mode (C) and positive ion mode (D). (1) Neochlorogenic acid; (2) Chlorogenic acid; (3) Cryptochlorogenic acid; (4) Coffeic acid; (5) Luteolin-7*-O-*rutinoside; (6) Luteolin-7-*O*-glucoside; (7) Luteolin-7-*O*- glucuronside; (9) isochlorogenic acid B; (10) isochlorogenic acid A; (11) Apigenin-7-*O*-glucoside; (13) isochlorogenic acid C; (15) Diosmetin-7-*O*-glucoside; (16) Apigenin-7- *O*-6"-malonylglucoside; (18) Linarin.

**2. Supplementary Tables**

**Supplementary Table 1. Repeatability of the analytes in *C. morifolium* flower (n=9)**

| Level | Weight  (mg) | SSDMC- Percent Content (%) | | | | | ESM- Percent Content (%) | | | | | | |
| --- | --- | --- | --- | --- | --- | --- | --- | --- | --- | --- | --- | --- | --- |
|  |  | C9 | C10 | C11 | C13 | C18 | C2 | C6 | C9 | C10 | C11 | C13 | C18 |
| Low | 125.0 | 0.077 | 0.728 | 0.312 | 0.753 | 0.027 | 0.490 | 0.318 | 0.077 | 0.735 | 0.315 | 0.768 | 0.027 |
|  | 125.2 | 0.072 | 0.709 | 0.317 | 0.742 | 0.026 | 0.491 | 0.314 | 0.071 | 0.715 | 0.32 | 0.757 | 0.026 |
|  | 125.1 | 0.077 | 0.700 | 0.316 | 0.746 | 0.028 | 0.487 | 0.312 | 0.076 | 0.705 | 0.321 | 0.761 | 0.028 |
| Medium | 250.1 | 0.074 | 0.723 | 0.318 | 0.746 | 0.025 | 0.495 | 0.307 | 0.073 | 0.728 | 0.321 | 0.768 | 0.025 |
|  | 250.1 | 0.075 | 0.718 | 0.319 | 0.766 | 0.026 | 0.486 | 0.309 | 0.074 | 0.723 | 0.324 | 0.781 | 0.026 |
|  | 250.0 | 0.077 | 0.715 | 0.320 | 0.768 | 0.026 | 0.489 | 0.311 | 0.076 | 0.721 | 0.325 | 0.783 | 0.026 |
| High | 375.1 | 0.071 | 0.720 | 0.320 | 0.769 | 0.029 | 0.492 | 0.315 | 0.07 | 0.725 | 0.323 | 0.784 | 0.029 |
|  | 375.1 | 0.072 | 0.718 | 0.322 | 0.739 | 0.028 | 0.493 | 0.311 | 0.071 | 0.723 | 0.325 | 0.754 | 0.028 |
|  | 375.2 | 0.075 | 0.721 | 0.321 | 0.724 | 0.029 | 0.491 | 0.315 | 0.074 | 0.728 | 0.324 | 0.746 | 0.029 |
| RSD% |  | 3.2 | 1.1 | 1.0 | 2.0 | 5.4 | 0.6 | 1.1 | 3.4 | 1.2 | 1.0 | 1.8 | 5.4 |

**Supplementary Table 2. Repeatability of the analytes in *C. morifolium* flower**

| Level | Weight  (mg) | SSDMC- Percent Content (%) | | | | ESM- Percent Content (%) | | | | | |
| --- | --- | --- | --- | --- | --- | --- | --- | --- | --- | --- | --- |
|  |  | C9 | C10 | C13 | C18 | C2 | C6 | C9 | C10 | C13 | C18 |
| Low | 125.0 | 0.12 | 0.105 | 0.172 | 0.443 | 0.242 | 0.439 | 0.119 | 0.103 | 0.17 | 0.445 |
|  | 125.0 | 0.121 | 0.104 | 0.173 | 0.441 | 0.244 | 0.437 | 0.12 | 0.102 | 0.171 | 0.445 |
|  | 125.1 | 0.123 | 0.104 | 0.178 | 0.441 | 0.242 | 0.44 | 0.121 | 0.102 | 0.176 | 0.445 |
| Medium | 250.1 | 0.12 | 0.106 | 0.178 | 0.419 | 0.243 | 0.441 | 0.119 | 0.103 | 0.176 | 0.422 |
|  | 250.0 | 0.121 | 0.106 | 0.18 | 0.412 | 0.243 | 0.442 | 0.119 | 0.104 | 0.177 | 0.415 |
|  | 250.0 | 0.125 | 0.106 | 0.184 | 0.422 | 0.244 | 0.44 | 0.123 | 0.104 | 0.182 | 0.425 |
| High | 375.0 | 0.117 | 0.102 | 0.175 | 0.395 | 0.239 | 0.427 | 0.116 | 0.1 | 0.173 | 0.398 |
|  | 375.1 | 0.118 | 0.103 | 0.177 | 0.401 | 0.241 | 0.433 | 0.117 | 0.101 | 0.175 | 0.404 |
|  | 375.0 | 0.119 | 0.104 | 0.178 | 0.400 | 0.242 | 0.432 | 0.118 | 0.101 | 0.176 | 0.403 |
| RSD% |  | 1.9 | 1.3 | 2.0 | 4.6 | 0.6 | 1.2 | 1.9 | 1.3 | 2 | 4.5 |

**Supplementary Table 3. Intermediate Precision of analytes in *C. morifolium* flower (CM) and *C. indicum* flower (CI)-Different Days, Different Analysts (n=9)**

| **Sample** | **Level** | **Weight (mg)** | **C2**  **(%)** | **RSD%** | **C6**  **(%)** | **RSD%** | **C9**  **(%)** | **RSD%** | **C10**  **(%)** | **RSD%** | **C11**  **(%)** | **RSD%** | **C13**  **(%)** | **RSD%** | **C18**  **(%)** | **RSD%** |
| --- | --- | --- | --- | --- | --- | --- | --- | --- | --- | --- | --- | --- | --- | --- | --- | --- |
| **SSDMC** | | | | | | | | | | | | | | | | |
| **CM** | Days | 250.2 | 0.493 | 2.8 | 0.322 | 2.7 | 0.077 | 3.6 | 0.724 | 3.0 | 0.324 | 2.9 | 0.773 | 2.6 | 0.024 | 4.8 |
|  | Analysts | 250.1 | 0.489 | 0.7 | 0.318 | 1.3 | 0.075 | 2.6 | 0.716 | 1.2 | 0.320 | 1.6 | 0.762 | 1.5 | 0.023 | 3.8 |
| **CI** | Days | 250.1 | 0.235 | 1.3 | 0.116 | 1.8 | 0.1 | 1.7 | 0.428 | 1.8 | - | - | 0.165 | 1.9 | 0.451 | 1.9 |
|  | Analysts | 250.0 | 0.238 | 1.2 | 0.115 | 1.7 | 0.099 | 1.1 | 0.426 | 1.1 | - | - | 0.164 | 1.2 | 0.448 | 1.2 |
| **ESM** | | | | | | | | | | | | | | | | |
| **CM** | Days | 250.2 | 0.493 | 2.8 | 0.322 | 2.7 | 0.075 | 2.8 | 0.726 | 3.0 | 0.323 | 2.9 | 0.775 | 2.6 | 0.027 | 4.9 |
|  | Analysts | 250.1 | 0.489 | 0.7 | 0.318 | 1.3 | 0.073 | 2.7 | 0.718 | 1.2 | 0.32 | 1.2 | 0.765 | 1.4 | 0.026 | 2.7 |
| **CI** | Days | 250.1 | 0.235 | 1.3 | 0.116 | 1.8 | 0.101 | 1.1 | 0.428 | 1.5 | - | - | 0.165 | 1.8 | 0.445 | 1.9 |
|  | Analysts | 250.0 | 0.238 | 1.2 | 0.115 | 1.7 | 0.101 | 1.2 | 0.427 | 1.1 | - | - | 0.164 | 1.2 | 0.444 | 1.2 |

**Supplementary Table 4. Intermediate Precision of analytes in *C. morifolium* flower–Different Equipments and columns**

| Level | Weight (mg) | SSDMC- Percent Content (%) | | | | | ESM- Percent Content (%) | | | | | | |
| --- | --- | --- | --- | --- | --- | --- | --- | --- | --- | --- | --- | --- | --- |
|  |  | C9 | C10 | C11 | C13 | C18 | C2 | C6 | C9 | C10 | C11 | C13 | C18 |
| Agilent 1260 | Phenomenex* | 0.073 | 0.722 | 0.311 | 0.764 | 0.026 | 0.491 | 0.319 | 0.076 | 0.719 | 0.318 | 0.761 | 0.023 |
|  | Phenomenex | 0.071 | 0.717 | 0.323 | 0.747 | 0.026 | 0.481 | 0.311 | 0.075 | 0.709 | 0.310 | 0.744 | 0.022 |
| Shimadzu  20A | Phenomenex* | 0.076 | 0.701 | 0.318 | 0.742 | 0.025 | 0.476 | 0.311 | 0.074 | 0.693 | 0.310 | 0.739 | 0.022 |
|  | Phenomenex | 0.073 | 0.728 | 0.324 | 0.770 | 0.027 | 0.493 | 0.323 | 0.078 | 0.715 | 0.323 | 0.767 | 0.024 |
| RSD% |  | 2.8 | 1.6 | 1.9 | 1.8 | 3.6 | 1.7 | 2.0 | 2.4 | 1.6 | 2.0 | 1.8 | 3.6 |

Phenomenex* and Phenomenex were columns from the same model but different batches.

**Supplementary Table 5. Intermediate Precision of analytes in *C. indicum* flower–Different Equipments and columns**

| Level | Weight (mg) | SSDMC-Percent Content (%) | | | | | ESM- Percent Content (%) | | | | | | |
| --- | --- | --- | --- | --- | --- | --- | --- | --- | --- | --- | --- | --- | --- |
|  |  | C9 | C10 | C11 | C13 | C18 | C2 | C6 | C9 | C10 | C11 | C13 | C18 |
| Agilent 1260 | Phenomenex* | 0.102 | 0.43 | 0.176 | 0.457 | 0.102 | 0.248 | 0.114 | 0.1 | 0.429 | 0.175 | 0.461 | 0.248 |
|  | Phenomenex | 0.094 | 0.4 | 0.166 | 0.461 | 0.094 | 0.241 | 0.112 | 0.094 | 0.408 | 0.168 | 0.457 | 0.241 |
| Shimadzu  20A | Phenomenex* | 0.1 | 0.431 | 0.168 | 0.444 | 0.1 | 0.235 | 0.113 | 0.098 | 0.423 | 0.169 | 0.45 | 0.235 |
|  | Phenomenex | 0.099 | 0.42 | 0.162 | 0.437 | 0.099 | 0.234 | 0.113 | 0.098 | 0.42 | 0.161 | 0.441 | 0.234 |
| RSD% |  | 3.7 | 3.4 | 3.5 | 2.5 | 3.7 | 2.6 | 0.9 | 2.6 | 2.1 | 3.3 | 2 | 2.6 |

Phenomenex* and Phenomenex were columns from the same model but different batches

**Supplementary Table 6. The results of recovery tests of analytes in *C. morifolium* flower (n=9)**

| Analytes | Original  (ug) | Spiked  (ug) | ESM | | | SSDMC | | |
| --- | --- | --- | --- | --- | --- | --- | --- | --- |
|  |  |  | Found  (ug) | Recovery  (%) | RSD  % | Found  (ug) | Recovery  (%) | RSD  % |
| Chlorogenic  acid | 613.5 | 285.8 | 887.2 | 95.8 | 3.1 |  |  |  |
|  | 613.3 | 571.6 | 1177.9 | 98.8 |  |  |  |  |
|  | 614.0 | 857.4 | 1487.4 | 101.9 |  |  |  |  |
| luteolin-7*-O-*β-  D-glucoside | 386.9 | 196.5 | 578.4 | 97.5 | 1.4 |  |  |  |
|  | 386.8 | 393.0 | 775.4 | 98.9 |  |  |  |  |
|  | 387.2 | 589.4 | 978.4 | 100.3 |  |  |  |  |
| 3,4-di*-O-*  caffeoylquinic  acid | 92.6 | 48.2 | 139.4 | 96.9 | 1.7 | 138.8 | 95.8 | 3.1 |
|  | 92.6 | 96.5 | 188.2 | 99.1 |  | 187.9 | 98.8 |  |
|  | 92.7 | 144.7 | 237.8 | 100.2 |  | 240.1 | 101.9 |  |
| 3,5-di*-O-*  caffeoylquinic  acid | 906.4 | 449.2 | 1343.3 | 97.2 | 1.7 | 1344.4 | 97.5 | 1.4 |
|  | 906.2 | 898.5 | 1796.0 | 99.0 |  | 1794.8 | 98.9 |  |
|  | 907.2 | 1347.7 | 2263.4 | 100.6 |  | 2258.9 | 100.3 |  |
| Apigenin-7*-O-*  β-D-glucoside | 404.4 | 205.7 | 604.6 | 97.3 | 1.1 | 603.7 | 96.9 | 1.7 |
|  | 404.3 | 411.4 | 808.9 | 98.4 |  | 812.0 | 99.1 |  |
|  | 404.7 | 617.0 | 1018.3 | 99.4 |  | 1022.9 | 100.2 |  |
| 4,5-di*-O-*  caffeoylquinic  acid | 972.8 | 488.5 | 1432.8 | 94.2 | 1.7 | 1440.8 | 95.8 | 2.5 |
|  | 972.5 | 977.0 | 1913.1 | 96.3 |  | 1922.1 | 97.2 |  |
|  | 973.6 | 1465.6 | 2403.0 | 97.5 |  | 2448.0 | 100.6 |  |
| linarin | 31.3 | 15.0 | 45.6 | 95.3 | 2.6 | 45.8 | 96.4 | 1.6 |
|  | 31.3 | 30.1 | 60.8 | 98.0 |  | 60.9 | 98.4 |  |
|  | 31.3 | 45.1 | 76.5 | 100.3 |  | 76.1 | 99.4 |  |

**Supplementary Table 7. The results of recovery tests of analytes in *C. indicum* flower (n=9)**

| Analytes | Original  (ug) | Spiked  (ug) | ESM | | | SSDMC | | |
| --- | --- | --- | --- | --- | --- | --- | --- | --- |
|  |  |  | Found  (ug) | Recovery  (%) | RSD  % | Found  (ug) | Recovery  (%) | RSD  % |
| Chlorogenic  acid | 559.2 | 94.5 | 649.2 | 95.3 | 3.2 |  |  |  |
|  | 558.7 | 189.0 | 746.0 | 99.1 |  |  |  |  |
|  | 574.8 | 283.5 | 862.7 | 101.6 |  |  |  |  |
| luteolin-7*-O-*β-  D-glucoside | 302.5 | 135.0 | 431.9 | 95.9 | 4.4 |  |  |  |
|  | 302.2 | 270.0 | 579.7 | 102.7 |  |  |  |  |
|  | 311.0 | 405.0 | 733.2 | 104.3 |  |  |  |  |
| 3,4-di*-O-*  caffeoylquinic  acid | 151.7 | 65.8 | 214.7 | 95.8 | 4.6 | 216.8 | 99.0 | 3.8 |
|  | 151.6 | 131.5 | 287.0 | 103.0 |  | 290.5 | 105.2 |  |
|  | 156.0 | 197.3 | 362.2 | 104.5 |  | 365.8 | 106.4 |  |
| 3,5-di*-O-*  caffeoylquinic  acid | 127.4 | 81.0 | 207.0 | 98.4 | 3.2 | 207.5 | 98.0 | 3.7 |
|  | 127.3 | 162.0 | 295.5 | 103.9 |  | 296.9 | 104.2 |  |
|  | 130.9 | 243.0 | 384.4 | 104.3 |  | 385.2 | 104.6 |  |
| 4,5-di*-O-*  caffeoylquinic  acid | 545.7 | 367.5 | 904.7 | 97.6 | 2.3 | 895.3 | 96.5 | 2.0 |
|  | 545.5 | 735.0 | 1289.5 | 101.2 |  | 1279.7 | 99.4 |  |
|  | 561.2 | 1102.5 | 1685.2 | 101.9 |  | 1667.7 | 100.3 |  |
| linarin | 216.1 | 115.8 | 326.5 | 95.4 | 4.0 | 323.2 | 96.5 | 2.6 |
|  | 215.9 | 231.5 | 451.0 | 101.6 |  | 447.7 | 99.6 |  |
|  | 222.1 | 347.3 | 578.8 | 102.8 |  | 573.3 | 101.6 |  |

**Supplementary Table 8. The factors and levels in robustness test**

| No. | Factors | Normal | -1 level | +1 level |
| --- | --- | --- | --- | --- |
| 1 | Flow rate (FR) | 1.0ml/min | -0.1 ml/min | +0.1 ml/min |
| 2 | Wave length (WL) | 327nm | -2 nm | +2 nm |
| 3 | Injection volume (IV) | 20ul | -5ul | +5ul |
| 4 | Acid concentration | 0.1% HAC | -0.02% HAC | +HAC |
| 5 | Col.temp.( CT) | 25℃ | -2℃ | +2℃ |
| 6 | Time of gradient (TP) | 0/10/14/20/35/40/45/55 min | -1min | +1min |
| 7 | Ratio of organic phase(MP1~MP7) | 10/18/19/19/20/22/25/35% | -1% | 1% |

**Supplementary Table 9. Percent Content (%) of investigated components in *C. morifolium* flower by SSDMC method**

| No. | C_2_ | C_6_ | C_9_ | C_10_ | C_11_ | C_13_ | C_18_ | C_7_ | C_15_ | C_16_ | Caf- | Fla- |
| --- | --- | --- | --- | --- | --- | --- | --- | --- | --- | --- | --- | --- |
| H1 | 0.357 | 0.463 | 0.086 | 0.845 | 0.453 | 0.672 | 0.018 | 0.141 | 0.468 | 0.670 | 1.960 | 0.357 |
| H2 | 0.138 | 0.078 | 0.026 | 0.297 | 0.020 | 0.222 | 0.070 | 0.062 | 0.058 | 0.028 | 0.684 | 0.138 |
| H3 | 0.449 | 0.360 | 0.053 | 0.864 | 0.395 | 0.575 | 0.091 | 0.426 | 0.509 | 0.838 | 1.941 | 0.449 |
| H4 | 0.533 | 0.495 | 0.103 | 1.046 | 0.522 | 0.745 | 0.102 | 0.548 | 0.716 | 1.252 | 2.426 | 0.533 |
| H5 | 0.498 | 0.310 | 0.074 | 0.689 | 0.315 | 0.772 | 0.021 | 0.178 | 0.365 | 0.684 | 2.032 | 0.498 |
| H6 | 0.242 | 0.318 | 0.033 | 0.563 | 0.402 | 0.468 | 0.109 | 0.337 | 0.469 | 0.979 | 1.305 | 0.242 |
| H7 | 0.304 | 0.401 | 0.052 | 0.639 | 0.537 | 0.559 | 0.100 | 0.432 | 0.635 | 1.348 | 1.554 | 0.304 |
| H8 | 0.262 | 0.336 | 0.035 | 0.561 | 0.413 | 0.478 | 0.084 | 0.344 | 0.462 | 0.962 | 1.335 | 0.262 |
| H9 | 0.740 | 0.300 | 0.306 | 1.146 | 0.072 | 1.120 | 0.276 | 0.359 | 0.228 | 0.052 | 3.311 | 0.740 |
| H10 | 0.510 | 0.552 | 0.089 | 1.179 | 0.684 | 0.818 | 0.127 | 0.658 | 0.670 | 1.452 | 2.596 | 0.510 |
| H11 | 0.322 | 0.132 | 0.148 | 0.526 | 0.027 | 0.486 | 0.124 | 0.160 | 0.083 | 0.021 | 1.480 | 0.322 |
| H12 | 0.451 | 0.502 | 0.101 | 0.966 | 0.590 | 0.712 | 0.120 | 0.553 | 0.655 | 1.342 | 2.229 | 0.451 |
| H13 | 0.433 | 0.452 | 0.071 | 0.887 | 0.566 | 0.628 | 0.117 | 0.505 | 0.620 | 1.359 | 2.019 | 0.433 |
| H14 | 0.267 | 0.188 | 0.090 | 0.592 | 0.252 | 0.352 | 0.063 | 0.208 | 0.226 | 0.507 | 1.301 | 0.267 |
| H15 | 0.678 | 0.523 | 0.146 | 1.068 | 0.605 | 0.783 | 0.131 | 0.647 | 0.658 | 1.289 | 2.676 | 0.678 |
| H16 | 0.738 | 0.570 | 0.164 | 1.164 | 0.652 | 0.859 | 0.145 | 0.700 | 0.717 | 1.377 | 2.925 | 0.738 |
| H17 | 0.721 | 0.549 | 0.154 | 1.132 | 0.642 | 0.830 | 0.154 | 0.691 | 0.662 | 1.302 | 2.838 | 0.721 |
| H18 | 0.370 | 0.314 | 0.053 | 0.626 | 0.359 | 0.620 | 0.065 | 0.258 | 0.417 | 0.831 | 1.669 | 0.370 |
| Mean  ±sd | 0.450  ±0.183 | 0.384  ±0.147 | 0.102  ±0.067 | 0.833  ±0.268 | 0.421  ±0.212 | 0.652  ±0.210 | 0.109  ±0.057 | 0.409  ±0.205 | 0.482  ±0.213 | 0.909  ±0.491 | 2.036  ±0.688 | 2.714  ±1.229 |
| G1 | 0.425 | 0.393 | 0.059 | 0.760 | 0.095 | 0.508 | 0.000 | 0.226 | 0.350 | 0.147 | 1.751 | 1.210 |
| G2 | 0.653 | 0.114 | 0.127 | 1.400 | 0.586 | 0.272 | 0.370 | 0.240 | 0.185 | 1.867 | 2.451 | 3.362 |
| G3 | 0.395 | 0.360 | 0.044 | 0.619 | 0.078 | 0.469 | 0.000 | 0.359 | 0.322 | 0.147 | 1.527 | 1.266 |
| G4 | 0.524 | 0.395 | 0.063 | 0.932 | 0.077 | 0.603 | 0.000 | 0.039 | 0.425 | 0.165 | 2.122 | 1.101 |
| G5 | 0.783 | 0.166 | 0.190 | 2.075 | 0.788 | 0.396 | 0.559 | 0.033 | 0.238 | 2.033 | 3.445 | 3.817 |
| G6 | 0.736 | 0.128 | 0.152 | 1.668 | 0.661 | 0.347 | 0.378 | 0.046 | 0.177 | 1.690 | 2.902 | 3.079 |
| G7 | 0.798 | 0.146 | 0.222 | 1.432 | 0.884 | 0.426 | 0.426 | 0.000 | 0.217 | 2.309 | 2.878 | 3.982 |
| G8 | 0.242 | 0.047 | 0.073 | 0.466 | 0.275 | 0.143 | 0.139 | 0.143 | 0.060 | 0.679 | 0.923 | 1.344 |
| G9 | 0.613 | 0.304 | 0.024 | 0.814 | 0.323 | 0.092 | 0.029 | 0.000 | 0.807 | 1.473 | 1.543 | 2.936 |
| G10 | 0.457 | 0.516 | 0.054 | 0.620 | 0.109 | 0.646 | 0.000 | 0.301 | 0.300 | 0.101 | 1.777 | 1.327 |
| G11 | 0.440 | 0.393 | 0.059 | 0.793 | 0.094 | 0.538 | 0.000 | 0.344 | 0.330 | 0.137 | 1.829 | 1.297 |
| G12 | 0.215 | 0.242 | 0.031 | 0.379 | 0.048 | 0.282 | 0.000 | 0.246 | 0.095 | 0.030 | 0.907 | 0.662 |
| G13 | 0.501 | 0.393 | 0.063 | 0.850 | 0.083 | 0.623 | 0.000 | 0.051 | 0.418 | 0.166 | 2.037 | 1.111 |
| G14 | 0.486 | 0.420 | 0.061 | 0.711 | 0.072 | 0.645 | 0.000 | 0.016 | 0.452 | 0.144 | 1.902 | 1.104 |
| G15 | 0.381 | 0.112 | 0.054 | 0.932 | 0.549 | 0.129 | 0.176 | 0.098 | 0.140 | 1.316 | 1.496 | 2.390 |
| G16 | 0.456 | 0.454 | 0.062 | 0.766 | 0.105 | 0.588 | 0.000 | 0.062 | 0.453 | 0.191 | 1.872 | 1.266 |
| G17 | 0.546 | 0.205 | 0.076 | 1.047 | 1.373 | 0.284 | 0.237 | 0.027 | 0.106 | 1.218 | 1.952 | 3.166 |
| G18 | 0.735 | 0.148 | 0.209 | 1.365 | 0.851 | 0.404 | 0.424 | 0.120 | 0.207 | 2.143 | 2.713 | 3.894 |
| G19 | 0.383 | 0.304 | 0.045 | 0.714 | 0.075 | 0.343 | 0.000 | 0.099 | 0.294 | 0.159 | 1.485 | 0.931 |
| Mean  ±sd | 0.514  ±0.169 | 0.276  ±0.140 | 0.088  ±0.061 | 0.965  ±0.435 | 0.375  ±0.385 | 0.407  ±0.177 | 0.144  ±0.192 | 0.129  ±0.120 | 0.293  ±0.174 | 0.848  ±0.843 | 1.974  ±0.661 | 2.066  ±1.168 |

C2 was component 2. So did the others. T1: Total content of Caffeoylquinic acids (C_2_, C_9_, C_10_, C_13)_; T1: Total content of Flavone glycosides (C_6_, C_7_, C_11_, C_15_, C_16_, C_18_);

**Supplementary Table 10. Percent Content (%) of investigated components in *C. morifolium* flower by SSDMC method**

| No. | C_2_ | C_6_ | C_9_ | C_10_ | C_11_ | C_13_ | C_18_ | C_7_ | C_15_ | C_16_ | T1 | T2 |
| --- | --- | --- | --- | --- | --- | --- | --- | --- | --- | --- | --- | --- |
| K1 | 0.371 | 0.485 | 0.082 | 0.714 | 0.635 | 0.717 | 0.079 | 0.306 | 0.132 | 0.400 | 0.371 | 0.485 |
| K2 | 0.283 | 0.360 | 0.058 | 0.531 | 0.444 | 0.516 | 0.060 | 0.228 | 0.107 | 0.332 | 0.283 | 0.360 |
| K3 | 0.166 | 0.072 | 0.024 | 0.202 | 0.016 | 0.261 | 0.036 | 0.061 | 0.058 | 0.010 | 0.166 | 0.072 |
| K4 | 0.111 | 0.187 | 0.004 | 0.126 | 0.087 | 0.103 | 0.014 | 0.010 | 0.034 | 0.043 | 0.111 | 0.187 |
| K5 | 0.350 | 0.865 | 0.006 | 0.647 | 0.727 | 1.022 | 0.012 | 0.310 | 0.317 | 0.339 | 0.350 | 0.865 |
| K6 | 0.341 | 0.474 | 0.053 | 0.523 | 0.494 | 0.677 | 0.036 | 0.265 | 0.085 | 0.199 | 0.341 | 0.474 |
| K7 | 0.406 | 0.318 | 0.053 | 0.582 | 0.163 | 0.843 | 0.104 | 0.165 | 0.046 | 0.059 | 0.406 | 0.318 |
| K8 | 0.604 | 0.548 | 0.065 | 0.538 | 0.320 | 0.604 | 0.109 | 0.061 | 0.062 | 0.092 | 0.604 | 0.548 |
| K9 | 0.158 | 0.134 | 0.013 | 0.305 | 0.151 | 0.274 | 0.022 | 0.148 | 0.094 | 0.291 | 0.158 | 0.134 |
| K10 | 0.419 | 0.523 | 0.090 | 0.757 | 0.836 | 1.054 | 0.074 | 0.316 | 0.127 | 0.457 | 0.419 | 0.523 |
| K11 | 0.241 | 0.137 | 0.050 | 0.402 | 0.051 | 0.466 | 0.142 | 0.065 | 0.139 | 0.033 | 0.241 | 0.137 |
| K12 | 0.561 | 0.700 | 0.087 | 0.701 | 0.514 | 1.032 | 0.038 | 0.182 | 0.225 | 0.367 | 0.561 | 0.700 |
| K13 | 0.378 | 0.534 | 0.024 | 0.446 | 0.576 | 0.656 | 0.020 | 0.226 | 0.163 | 0.363 | 0.378 | 0.534 |
| K14 | 0.575 | 0.582 | 0.158 | 1.094 | 0.649 | 1.336 | 0.104 | 0.652 | 0.388 | 1.094 | 0.575 | 0.582 |
| K15 | 0.292 | 0.380 | 0.068 | 0.584 | 0.482 | 0.642 | 0.058 | 0.243 | 0.112 | 0.357 | 0.292 | 0.380 |
| K16 | 0.508 | 0.518 | 0.104 | 1.054 | 0.610 | 0.961 | 0.085 | 0.450 | 0.349 | 0.972 | 0.508 | 0.518 |
| Mean  ±sd | 0.360  ±0.150 | 0.426  ±0.218 | 0.058  ±0.041 | 0.575  ±0.263 | 0.422  ±0.259 | 0.698  ±0.333 | 0.062  ±0.039 | 0.231  ±0.162 | 0.152  ±0.110 | 0.338  ±0.310 | 0.360  ±0.150 | 0.426  ±0.218 |

C2 was component 2. So did the others. T1: Total content of Caffeoylquinic acids (C_2_, C_9_, C_10_, C_13)_; T1: Total content of Flavone glycosides (C_6_, C_7_, C_11_, C_15_, C_16_, C_18_).

**Supplementary Table 11. Content (%) of investigated components in** ***C. indicum* flower by SSDMC method**

| No. | C_2_ | C_6_ | C_9_ | C_10_ | C_13_ | C_18_ | C_7_ | T1 | T2 |
| --- | --- | --- | --- | --- | --- | --- | --- | --- | --- |
| Y1 | 0.326 | 0.355 | 0.034 | 0.531 | 0.319 | 0.120 | 0.243 | 1.211 | 0.718 |
| Y2 | 0.342 | 0.149 | 0.176 | 0.508 | 0.235 | 0.631 | 0.356 | 1.260 | 1.137 |
| Y3 | 0.566 | 0.127 | 0.104 | 0.547 | 0.323 | 0.134 | 0.038 | 1.540 | 0.299 |
| Y4 | 0.384 | 0.078 | 0.060 | 0.524 | 0.282 | 0.033 | 0.032 | 1.249 | 0.144 |
| Y5 | 0.574 | 0.159 | 0.067 | 0.553 | 0.238 | 0.221 | 0.046 | 1.433 | 0.426 |
| Y6 | 0.093 | 0.012 | 0.010 | 0.177 | 0.042 | 0.344 | 0.000 | 0.321 | 0.357 |
| Y7 | 0.197 | 0.123 | 0.050 | 0.143 | 0.098 | 0.383 | 0.146 | 0.488 | 0.651 |
| Y8 | 0.066 | 0.018 | 0.003 | 0.149 | 0.031 | 1.450 | 0.000 | 0.249 | 1.468 |
| Y9 | 0.265 | 0.255 | 0.066 | 0.276 | 0.227 | 0.019 | 0.300 | 0.834 | 0.573 |
| Y10 | 0.311 | 0.307 | 0.071 | 0.321 | 0.284 | 0.024 | 0.343 | 0.988 | 0.674 |
| Y11 | 0.425 | 0.253 | 0.084 | 0.344 | 0.273 | 0.332 | 0.246 | 1.126 | 0.831 |
| Y12 | 0.119 | 0.047 | 0.034 | 0.144 | 0.052 | 0.351 | 0.050 | 0.348 | 0.448 |
| Y13 | 0.201 | 0.034 | 0.018 | 0.377 | 0.107 | 1.508 | 0.015 | 0.704 | 1.557 |
| Y14 | 0.292 | 0.112 | 0.027 | 0.136 | 0.079 | 0.287 | 0.098 | 0.533 | 0.496 |
| Y15 | 0.161 | 0.070 | 0.022 | 0.212 | 0.088 | 1.283 | 0.061 | 0.483 | 1.413 |
| Y16 | 0.186 | 0.129 | 0.030 | 0.083 | 0.118 | 0.108 | 0.058 | 0.417 | 0.295 |
| Y17 | 0.392 | 0.067 | 0.027 | 0.505 | 0.173 | 1.984 | 0.027 | 1.097 | 2.078 |
| Y18 | 0.303 | 0.151 | 0.066 | 0.263 | 0.177 | 0.153 | 0.120 | 0.810 | 0.424 |
| Y19 | 0.285 | 0.106 | 0.053 | 0.391 | 0.132 | 0.808 | 0.104 | 0.861 | 1.018 |
| Y20 | 0.091 | 0.089 | 0.007 | 0.089 | 0.058 | 0.096 | 0.046 | 0.245 | 0.231 |
| Y21 | 0.331 | 0.170 | 0.058 | 0.314 | 0.184 | 0.065 | 0.099 | 0.887 | 0.333 |
| Y22 | 0.275 | 0.053 | 0.024 | 0.308 | 0.131 | 1.310 | 0.028 | 0.738 | 1.390 |
| Y23 | 0.110 | 0.052 | 0.039 | 0.156 | 0.070 | 0.224 | 0.062 | 0.375 | 0.338 |
| Y24 | 0.396 | 0.232 | 0.020 | 0.554 | 0.215 | 1.328 | 0.136 | 1.184 | 1.696 |
| Y25 | 0.049 | 0.057 | 0.032 | 0.044 | 0.041 | 0.023 | 0.095 | 0.166 | 0.175 |
| Y26 | 0.174 | 0.022 | 0.016 | 0.223 | 0.117 | 0.750 | 0.000 | 0.530 | 0.772 |
| Y27 | 0.104 | 0.021 | 0.005 | 0.140 | 0.053 | 1.487 | 0.000 | 0.302 | 1.508 |
| Y28 | 0.079 | 0.057 | 0.030 | 0.086 | 0.073 | 0.217 | 0.084 | 0.269 | 0.358 |
| Y29 | 0.507 | 0.109 | 0.018 | 0.721 | 0.119 | 1.446 | 0.038 | 1.365 | 1.593 |
| Y30 | 0.320 | 0.266 | 0.013 | 0.309 | 0.181 | 0.527 | 0.130 | 0.824 | 0.923 |
| Y31 | 0.314 | 0.129 | 0.058 | 0.202 | 0.109 | 0.250 | 0.158 | 0.683 | 0.538 |
| Y32 | 0.536 | 0.093 | 0.030 | 1.194 | 0.156 | 1.542 | 0.014 | 1.916 | 1.650 |
| Y33 | 0.508 | 0.092 | 0.037 | 1.394 | 0.173 | 1.471 | 0.016 | 2.112 | 1.580 |
| Mean  ±sd | 0.281  ±0.153 | 0.121  ±0.088 | 0.042  ±0.034 | 0.361  ±0.298 | 0.150  ±0.086 | 0.634  ±0.607 | 0.097  ±0.099 | 0.835  ±0.495 | 0.851  ±0.555 |

C2 was component 2. So did the others. T1: Total content of Caffeoylquinic acids (C_2_, C_9_, C_10_, C_13)_; T1: Total content of Flavone glycosides (C_6_, C_7_, C_11_, C_15_, C_16_, C_18_).
